# The effects of supplementation of probiotics, prebiotics, or synbiotics on patients with non-alcoholic fatty liver disease: A meta-analysis of randomized controlled trials

**DOI:** 10.3389/fnut.2022.1024678

**Published:** 2022-10-25

**Authors:** Wenmin Xing, Wenyan Gao, Xiaoling Lv, Zhenlei Zhao, Genxiang Mao, Xiaoyan Dong, Zuyong Zhang

**Affiliations:** ^1^Zhejiang Provincial Key Laboratory of Geriatrics, Department of Geriatrics, Zhejiang Hospital, Hangzhou, China; ^2^School of Pharmacy, Hangzhou Medical College, Hangzhou, China; ^3^College of Pharmacy and Traditional Chinese Medicine, Jiangsu College of Nursing, Huaian, China; ^4^The Third People’s Hospital of Hangzhou, Hangzhou, China

**Keywords:** non-alcoholic fatty liver disease, probiotics, prebiotics, liver enzymes, lipid profile, resistance, meta-analysis

## Abstract

**Background:**

Non-alcoholic fatty liver disease (NAFLD) is the most prevalent chronic liver disease. Research on the efficacy of probiotics, prebiotics, and synbiotics on NAFLD patients continues to be inconsistent. The purpose of this study is to evaluate the effectiveness of these microbial therapies on NAFLD.

**Methods:**

Eligible randomized-controlled trials reporting the effect of probiotics, prebiotics, or synbiotics in NAFLD were searched in PubMed, Web of Science, Embase, Google scholar, and CNKI databases from 2020 to Jul 2022. The changes in the outcomes were analyzed using standard mean difference (SMD) and 95% confidence intervals (CIs) with a random- or fixed-effects model to examine the effect of microbial therapies. Subgroup analysis, influence and publication bias analysis were also performed. The quality of the eligible studies was evaluated using the Cochrane Risk of Bias Tool.

**Results:**

Eleven studies met the inclusion criteria involving 741 individuals. Microbial therapies could improve liver steatosis, total cholesterol (TC), triglyceride (TG), low-density lipoprotein (LDL-c), alanine aminotransferase (ALT), alkaline phosphatase (ALP), glutamyl transpeptidase (GGT), and homeostasis model assessment-insulin resistance (HOMAI-R) (all *P* < 0.05). But microbial therapies could not ameliorate body mass index (BMI), energy, carbohydrate, fat intake, fasting blood sugar, HbA1c, insulin, high-sensitivity C-reactive protein (hs-CRP), and hepatic fibrosis of patients with NAFLD.

**Conclusion:**

Probiotics, prebiotics, and synbiotics supplementation can potentially improve liver enzymes, lipid profiles, and liver steatosis in patients with NAFLD.

## Introduction

Non-alcoholic fatty disease (NAFLD) is the most prevalent liver disease worldwide due to an increase inobesity and diabetes, especially in western developed countries (1). NAFLD has now become a public health issue, following globalization and rejuvenation trends (2). In addition to deteriorating into non-alcoholic steatohepatitis (NASH), liver cirrhosis, and hepatocellular carcinoma, NAFLD can result in cardiovascular, cerebrovascular diseases, and metabolic syndrome (MS) (3). Various researchers have investigated the complex pathogenesis of NAFLD. In addition to insulin resistance, oxidative stress, inflammatory mediators, and cytokines factors (4), intestinal flora was also involved in the progression of NAFLD identified in both human and animal models (1). Changes in the microbiome can result in intestinal dyskinesia and inflammation, and affect the severity of NAFLD (5). The dysbiosis of intestinal flora may cause an increase in the permeability rate of the small intestine and disrupt gut barrier integrity. Consequently, toxic bacterial metabolic by-products derived from the gut microbiota and endotoxins will continuously enter into the circulation and impair liver function (6, 7). Therefore, efficient interventions and modifying the gut microbiota to restore intestinal microbial diversity may be novel methods to improve NAFLD.

Several researchers have investigated the effectiveness of microbial therapies for NAFLD (8–12). Probiotics, for instance, have been confirmed as a group of active microorganisms that are beneficial to the host by colonizing the human gut and reproductive system to improve the host’s imbalanced microbiota (13). It has been reported that (14) probiotic supplementation can reduce the production of pathogenic bacteria by absorbing endotoxin, improve the microecological balance and reduce the production and entry of harmful substances into the liver, thereby preventing and alleviating the pathological process of NAFLD. Prebiotics are additional effective interventions through dietary supplements of indigestible food ingredients that improve host health by selectively stimulating the growth and activity of bacteria in one or a small number of colonies. Prebiotics can indirectly affect the human body by influencing the activity of probiotics (13). The combination of probiotics and prebiotics is known as synbiotis (15, 16). Previous studies have investigated the efficacy of these treatments for NAFLD (17–23). Nonetheless, some meta-analysis studies only included a few published studies (17, 19, 21–23), and some of these only addressed the efficacy of probiotics (19, 21, 23). In addition, all included studies were outdated, which may result in the inaccuracy of pooled analysis results.

A healthy lifestyle has been suggested as the most prevalent intervention to mitigate or reverse NAFLD pathogenesis (24), such as, weight reduction through diet and exercise (24). Therefore, recent reports on probiotics, prebiotics, or symbiotics treatment also investigated the change in the intake of energy, carbohydrate, and total fat compared between the initial point and intervention endpoint (8, 9, 15, 18). In addition, fasting blood sugar (FBS), glycated hemoglobin (HbA1c), insulin, homeostasis model assessment-insulin resistance (HOMAI-R), lipid profiles, systematic inflammation, and liver enzymes, were also evaluated. No meta-analysis study has evaluated the effect of microbial therapies on all the following parameters among NAFLD patients: dietary change, lipid profiles, glucose homeostasis parameters, systematic inflammation, liver enzymes, and hepatic features (fibrosis and steatosis). This study aims to comprehensively evaluate the efficacy of probiotics, prebiotics, and synbiotics in NAFLD patients in light of recent studies. In study, the primary outcomes, including intake of energy, carbohydrate, and total fat, blood sugar homeostasis, lipid profiles, liver enzymes, systematic inflammation, hepatic fibrosis, and steatosis, were extracted from the included studies, followed by subgroup analyses and publication bias assessment. For each intervention and control group, the changes in mean and standard deviation (SD) values on the baseline and final points were computed. Continuous variables (the changes in mean and SD of the outcomes) were analyzed using standard mean difference (SMD) and 95% confidence intervals (CIs).

## Methods

This present meta-analysis was performed according to the Systematic and Meta-analytical Preferred Reporting Project (PRISMA) statement (25).

### Search strategy

Two authors performed independently searched the electronic database of Pubmed, PMC, ISI Web of Science, Embase, Cochrane Library, and Chinese National Knowledge Infrastructure (CNKI). These eligible articles were published in English and Chinese between January 2020 and Jul 2022. The following keywords used for the literature search were: “probiotic,” “prebiotic,” “dietary fiber,” “symbiotic,” “symbiotic,” “non-alcoholic fatty liver disease,” “NAFLD,” “fatty liver,” “non-alcoholic steatohepatitis,” or “NASH.” In addition, studies were limited to randomized controlled trials (RCTs). To avoid missing the eligible studies, the references cited in eligible studies were also manually searched.

### Inclusion and exclusion criteria

Two authors independently read the titles, abstracts, and full text of the articles that matched the inclusion criteria. The inclusion criteria were as follows: (1) a randomized controlled trial (RCT) was designed; (2) patients were diagnosed with NAFLD; (3) the effects of probiotics, prebiotics, or synbiotics were evaluated in NAFLD patients and control subjects; (4) studies reported data both on baseline and end of intervention for the outcomes: BMI, body fat, dietary intake of energy, carbohydrate, and fat, FBS, HbA1c, insulin (INS), HOMA-IR, high-sensitivity C-reactive protein (hs-CRP), total cholesterol (TC), low-density lipoprotein-cholesterol (LDL-C), high-density lipoprotein-cholesterol (HDL-C), TAC, triglycerides (TG), alanine aminotransferase (ALT), aspartate aminotransferase (AST), and gamma-glutamyl transferase (γ-GGT), hepatic steatosis and fibrosis; (5) studies language were districted in English or Chinese. These studies will be excluded if they meet any one of the following exclusion criteria: (1) hepatic steatosis or fibrosis in patients were caused by autoimmune hepatitis, hepatitis, liver cancer, or other factors; (2) the study design was not RCT; (3) the study didn’t provide the baseline and final outcome data; (4) the study was duplicated.

### Data extraction and quality assessment

Two authors independently extracted the data from these included studies, and any disagreements were resolved through further discussion. The following information was extracted from each study: the first author, the publication data, the subject’s ethnicity, the number of case and control groups, ages of case and control subjects, intervention, follow-up duration, and outcomes. The mean and SD values at the baseline and the endpoint were directly extracted or calculated from the provided data provided by each study.

The risk of bias in each RCT was assessed by the Cochrane Collaboration’s tool in RevMan5.4 software^1^ (26, 27). Briefly, the risk of bias includes seven domains: random sequence generation, allocation concealment, blinding of participants and personnel, blinding of outcome assessment, incomplete outcome data, selective reporting, and other types of bias (27). Each study was evaluated by two authors, and any disagreement was further discussed with the third author.

### Data statistical analysis

This meta-analysis estimated the liver-related changes, including subject metabolic characteristics (BMI), dietary components’ intake (energy, carbohydrate, and total fat), blood glucose homeostasis assessments (FBS, HbA1c, insulin, and HOMA-IR), and hs-CRP, hepatic features (steatosis and fibrosis) and liver enzyme levels, lipid profiles (changes in TC, TG, LDL-C, and HDL-C).

The differences in these continuous outcome indexes were calculated and analyzed using SMD and CIs from the baseline to the final points. A *p*-value < 0.05 was considered statistically significant. The heterogeneity of this meta-analysis was also evaluated by χ^2^ and *I*^2^ tests. *I*^2^ values of 25%, 50%, and 75% indicated a slight, moderate, and high level of heterogeneity, respectively. If the statistical heterogeneity *I*^2^ was ≥ 50% among the studies, the random effect model would be used to estimate the pooled analysis. Otherwise, the fixed-effect model will be used in the combined analysis. All the analyses were conducted using the Stata 12.0 (Stata Corp, College Station, TX, USA) software.

### Assessment of publication bias and sensitivity analysis

Egger’s test and Begg’s test were used to visually assess publication bias through funnel plots. In addition, we also performed a sensitivity analysis to determine the consistency of the results. The evaluation of funnel plots and sensitivity analysis were performed using the software Stata 12.0 (Stata Corp, College Station, TX, USA). In case of significant funnel asymmetry or any outside of the upper or the lower limit of the sensitivity analysis, we further performed subgroup analysis based on the characteristics of the study, including the difference in ethnicity, intervention, and others.

## Results

### Clinical characteristics of the included studies and patients

**FIGURE 1:**
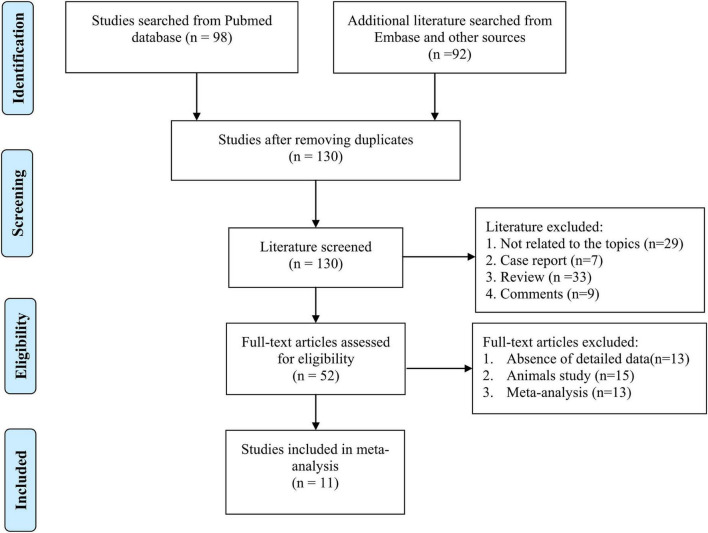
The flowchart diagram for literature selecting process from database.

According to the searched results, there were a total of 192 records, 62 of which are duplicates that need to be removed. Then, we removed 78 articles that did not meet the inclusion criteria based on the titles and abstracts. After reading the full text of these articles, another 41 articles were removed due to a lack of detailed data to calculate the change of designate indexes, such as, BMI, liver features, insulin, and others. Finally, we included 11 (8–12, 15, 16, 18, 28–30) different RCTs that met all the inclusion with the following outcomes: BMI (*n* = 9), energy, carbohydrate, and total fat intake (*n* = 4), FBS (*n* = 8), HbA1c (*n* = 3), insulin (*n* = 5), HOMA-IR (*n* = 5), hs-CRP (*n* = 9), hepatic features [steatosis (*n* = 4), and fibrosis (*n* = 4)], ALP (*n* = 3), ALT (*n* = 10), AST (*n* = 10), GGT (*n* = 8), TC (*n* = 10), TG (*n* = 10), LDL-C (*n* = 9), HDL-C (*n* = 9). The details of the flowchart diagram are shown in Figure 1.

The clinical characteristics of the studies and subjects were described in Table 1. These studies included 741 individuals (392 patients with NAFLD, and 349 healthy individuals). The included studies were published from Jan 2020 to Jul 2022, and conducted in Asia (9, 28), North America (11), and Europe (8, 10, 12, 15, 16, 18, 29, 30), respectively. There are eight studies with the probiotics intervention, two with synbiotics intervention, and one with prebiotics intervention for patients with NAFLD. In addition, some studies presented data on multiple different intervention groups. We, therefore, treated these groups as independent investigations. One of the included trials involved children with NAFLD, while the remaining trials involved adults with NAFLD. The patient and control sample sizes ranged from 16 to 70 individuals. The median follow-up duration of these studies ranged from 2.5 to 14 months.

**TABLE 1:** The clinical characters of the included patients and studies.

References	Ethnicity	Study design	Subjects No. (case/control)	Age of case (years)	Intervention	Follow-up duration	Outcomes
Derosa et al. (11)	Caucasian	RCT	30/30	>18 years	Patients were randomized to take placebo or VSL^#^3R, 2 sachets/day	3 months	No change in BMI, FPG, TC, LDL-C, HDL-C, and ADN. Tg, Hs-CRP, GGT, Transaminases, AST/ALT ratio, and HSI decreased
Mohamad Nor et al. (9)	Asian	RCT	17/22	53.44 (14.13)	Probiotics containing six different Lactobacillus and Bifidobacterium species at a concentration of 30 billion CFU were used for patients	6 months	No changes in hepatic steatosis fibrosis levels, AST, total cholesterol, Tg, glucose, CD4^+^ T lymphocytes. CD8^+^ T lymphocytes, ZO-1 decreased.
Crommen et al. (10)	European	RCT	25/23	38 (79)	The patients were treated with a specifically tailored multistrain probiotic powder	3 months	ASAT, Tg, liver fibrosis score, the visceral adiposity index decreased.
Rodrigo et al. (12)	European	RCT	43/41	11.3 (1.9)	Probiotics (BioKult 14 strain capsule) for 6 months	6 months	Tg, AST, ALT, AST/ALT ratio, and alkaline phosphatase decreased.
Chong et al. (30)	European	RCT	19/16	57 (8)	Patients were randomly allocated to take 2 sachets VSL^#^3 probiotic.	2.5 months	No significantly improve insulin resistance, endothelial dysfunction, oxidative stress, inflammation, or liver injury.
Kavyani et al. (8)	European	RCT	18/18	43.72 (4.49)	The intervention group received fat (∼20 g) as CSO with 10 g/d resistant dextrin and the placebo group received a similar amount of CSO and maltodextrin.	3 months	Insulin concentration, HOMA-IR, hs-CRP, endotoxin, cortisol, GHQ, DASS, and MDA decreased, TAC and superoxide dismutase increased.
Scorletti et al. (16)	European	RCT	55/49	50.8 (12.6)	Patients received fructo-oligosaccharides with a degree of polymerization < 10 at 4 g/twice a day.	14 months	No reduction in liver fat, patients had higher Bifidobacterium and Faecalibacterium, and lower in Oscillibacter and Alistipes.
Cai et al. (28)	Asian	RCT	70/70	46.13 (12.72)	Live Combined Bifidobacterium, Lactobacillus, and Enterococcus Powder was given orally, 1 g/time, 2 times/d for the patients.	3 months	ALT, AST, GGT, TC, TG, HOMA-IR, NAS, and conditions of fecal flora in the observation group were better than those in the control group.
Abhari et al. (15)	European	RCT	23/22	47.7 (11.4)	Patients received either synbiotic or placebo capsules for 12 weeks.	3 months	Decrease AST, GST, TNF-α, NF-kB, and hepatic steatosis
Behrouz et al. (18)	European	RCT	30/29/30	38.46 (7.11)	The patients were randomly assigned to the three groups: A (probiotic capsules and placebo of prebiotic), B (prebiotic powder and placebo of probiotic), and C (placebo of probiotic and placebo of prebiotic)	3 months	Decreased triglyceride, ALT, AST, GGT, hsCRP, and alkaline phosphatase. Triglyceride, LDL-C, ALT, AST, and GGT differed in prebiotic group in comparison to the placebo.
Sadrkabir et al. (29)	European	RCT	33/28	43.26 (11.42)	The intervention group received daily 2 capsules of 500 mg of GeriLact.	2 months	Decrease ALT, AST, cholesterol levels, no significant changes in FBS, triglycerides, LDL, and HDL; fatty liver grade was improved by 63.6% in the intervention group

Age of case was expressed as mean (SD).

BMI, Body mass index; FPG, fasting plasma glucose; TC, total cholesterol; LDL-c, low-density lipoprotein-cholesterol; HDL-c, high-density lipoprotein-cholesterol; AND, adiponectin; TG, Triglycerides; Hs-CRP, high-sensitivity C-reactive protein; AST, Aspartate aminotransferase; ALT, alanine aminotransferase; HIS, hepatic steatosis index; LBP, lycium barbarum polysaccharide; TAC, total antioxidant capacity; MDA, malondialdehyde; CSO, Camelina sativa oil; DASS, depression, anxiety and stress scale; GHQ, general health questionnaire; GGT, γ-glutamyltransferase; TNF-α, tumor necrosis factor-α; NF-kB, nuclear factor-kB.

The risk bias of the included RCTs was shown in Supplementary Figure 1. These RCTs were well designed in terms of random sequence generation. Only one study was considered to have a substantial risk of bias. The majority of the studies did not discuss the blinding of outcome assessment. Regarding the blinding of participants and personnel, four studies reported an unclear risk of bias. Six RCTs had a low risk of bias regarding incomplete outcome data. Four studies showed a high risk of biased response to selectively reporting results.

### Body mass index and dietary intake

**FIGURE 2:**
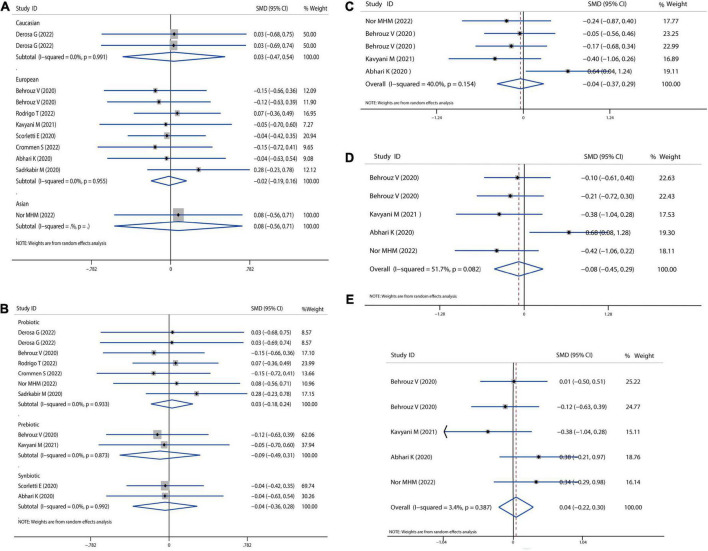
Forest plot of the effect of microbiota therapies on BMI and dietary intake in patients with NAFLD. **(A)** The effect of microbiota therapies on BMI based on different ethnicity. **(B)** The effect of microbiota therapies on BMI based on different interventions. **(C)** The effect of microbiota therapies on the intake of energy. **(D)** The effect of microbiota therapies on the intake of carbohydrates. **(E)** The effect of microbiota therapies on the intake of fat. For each study, the estimated mean changes and the 95% CI are plotted with a diamond and a horizontal line, respectively.

Firstly, we examined the effects of probiotics, prebiotics, or symbiotics on the BMI across all study populations. We observed no significant difference in absolute changes for BMI (SMD = −0.01, 95% CI = −0.17 to 0.15, *I*^2^ = 0.0%, *P* = 0.995), but there was a decreasing trend following the intervention. Subgroup analysis based on different ethnicity and intervention showed that there was no significant difference between the microbial treatment responses of Caucasian, European, or Asian, as revealed in Figures 2A,B. Sensitivity analysis showed that there was no data outside of the lower or upper limit, and the effect estimates were robust and reliable. According to Begg’s test (*P* = 0.586) and Egger’s test (*P* = 0.996), there was no publication bias, as shown in Supplementary Data Sheet 1.

Then, we evaluated the effects of probiotics, prebiotics, or synbiotics on the dietary habits in all populations under study. The absolute mean and SD of dietary intakes are shown in Figures 2C–E. Compared to the intervention group and placebo group, intake of energy, carbohydrates, and fat was reduced, but the reduction was not significantly different.

### Glucose homeostasis

**FIGURE 3:**
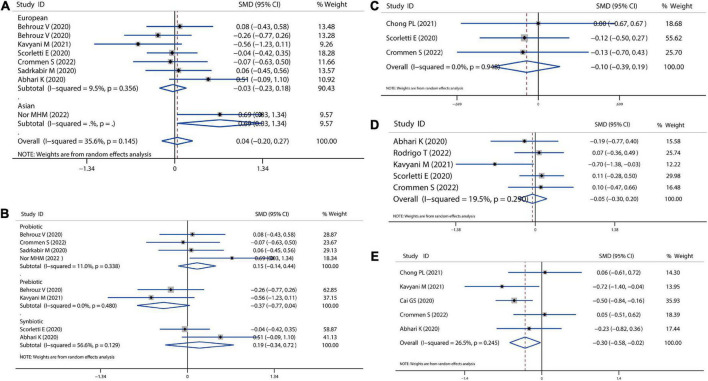
Forest plot of the effect of microbiota therapies on glucose homeostasis in patients with NAFLD. **(A)** The effect of microbiota therapies on FBS based on different ethnicity. **(B)** The effect of microbiota therapies on FBS based on different interventions. **(C)** The effect of microbiota therapies on the intake of energy. **(D)** The effect of microbiota therapies on the level of HbA1c. **(E)** The effect of microbiota therapies on the change of HOMAI-R. For each study, the estimated mean changes and the 95% CI are plotted with a diamond and a horizontal line, respectively.

We also calculated the mean difference in absolute changes for FBS, HbA1c, insulin, and HOMAI-R after probiotics, prebiotics, or synbiotics administration. There was no significant reduction in the overall analysis for FBS, although there was a slight reduction in the European population (SMD = −0.03, 95% CI = −0.23 to 0.18, *I*^2^ = 9.5%, *P* = 0.785) (Figure 3A). In addition, there was likely higher reduction for FBS by prebiotics than probiotics administration (prebiotic: SMD = −0.37, 95% CI = −0.77 to 0.04, *I*^2^ = 0.0%, *P* = 0.075; probiotics: SMD = 0.15, 95% CI = −0.14 to 0.44, *I*^2^ = 11.0%, *P* = 0.316; Figure 3B). Moreover, we observed a reduction trend toward lower levels of HbA1c (SMD = −0.10 95% CI = −0.39 to 0.19, *I*^2^ = 0.0%, *P* = 0.497; Figure 3C) and insulin (SMD = −0.05, 95% CI = −0.30 to 0.20, *I*^2^ = 19.5%, *P* = 0.702; Figure 3D), however, this was not significant. A significant decrease in HOMAI-R (SMD = −0.30, 95% CI = −0.58 to −0.02, *I*^2^ = 26.5%, *P* = 0.034; Figure 3E) was observed. Sensitivity analysis for FBS showed no data outside of the lower or upper limit, and the effect estimates were robust and reliable. There was also no publication bias according to Begg’s test (*P* = 0.621) and Egger’s test (*P* = 0.636), as shown in Supplementary Data Sheet 1.

### Lipid profile

**FIGURE 4:**
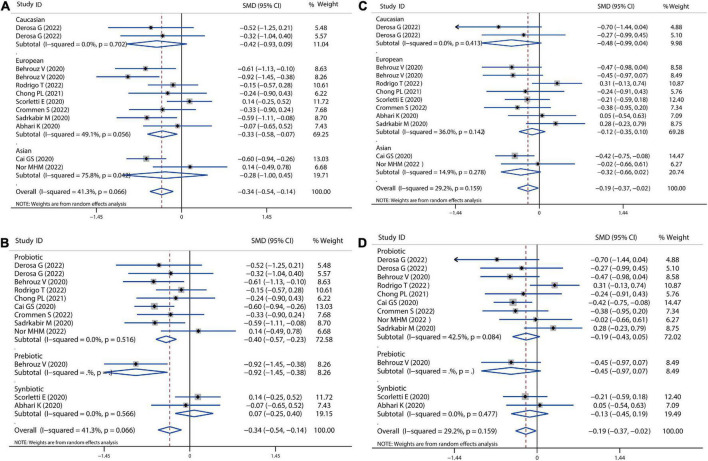
Forest plot of the effect of microbiota therapies on TC and TG in patients with NAFLD. **(A)** The effect of microbiota therapies on TC based on different ethnicity. **(B)** The effect of microbiota therapies on TC based on different interventions. **(C)** The effect of microbiota therapies on TG based on different ethnicity. **(D)** The effect of microbiota therapies on TG based on different interventions. For each study, the estimated mean changes and the 95% CI are plotted with a diamond and a horizontal line, respectively.

**FIGURE 5:**
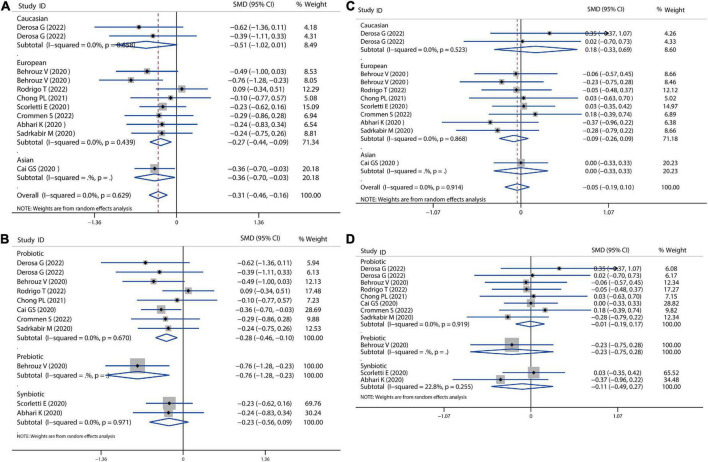
Forest plot of the effect of microbiota therapies on LDL-c and HDL-c in patients with NAFLD. **(A)** The effect of microbiota therapies on LDL-c based on different ethnicity. **(B)** The effect of microbiota therapies on LDL-c based on different interventions. **(C)** The effect of microbiota therapies on HDL-c based on different ethnicity. **(D)** The effect of microbiota therapies on HDL-c based on different interventions. For each study, the estimated mean changes and the 95% CI are plotted with a diamond and a horizontal line, respectively.

A meta-analysis of serum lipid profiles was conducted on the included studies that reported TC, TG, LDL-c, and HDL-c. Overall, results showed that probiotics, prebiotics, or synbiotics therapies significantly reduced TC (SMD = −0.34, 95% CI = −0.54 to −0.14, *I*^2^ = 41.3%, *P* < 0.001) (Figure 4A). According to the subgroup analysis by ethnicity, this reduction in Caucasian, European, and Asian populations was 0.42, 0.33, and 0.28, respectively. Meantime, individually probiotic treatment had a more reduced effect on TC (SMD = −0.40, 95% CI = −0.57 to −0.23, *I*^2^ = 0.0%, *P* < 0.001) than other treatment, while synbiotics treatment did not decrease TC (Figure 4B). Individually probiotics, prebiotics, or synbiotics treatment could also combined decrease the TG level of patients with NAFLD (SMD = −0.19, 95% CI = −0.37 to −0.03, *I*^2^ = 29.2%, *P* < 0.001). The treatment effect was similar to the reduction of TC (Figures 4C,D). Then, we combined the effects of the three treatments on LDL-c and HDL-c. Results showed that the three treatments significantly decreased the level of LDL-c (SMD = −0.31, 95% CI = −0.46 to −0.16, *I*^2^ = 0.0%, *P* < 0.001) and slightly decreased the level of HDL-c (SMD = −0.05, 95% CI = −0.19 to −0.10, *I*^2^ = 0.0%, *P* = 0.055), shown as in Figure 5. Analysis by ethnicity showed that all three treatments could decrease the level of LDL-c in the Caucasian, European, and Asian populations by a SMD value of −0.51, −0.27, and −0.36, respectively (Figure 5A). Analysis by treatment type showed that probiotics decreased the level of LDL-c (SMD = −0.28, 95% CI = −0.46 to −0.10, *I*^2^ = 0.0%, *P* = 0.002), but synbiotics did not (*P* = 0.157) (Figure 5B). Neither probiotics, prebiotics, nor symbiotic could change the level of HDL-c in any population, as shown in Figures 5C,D.

Sensitivity analysis showed no data outside of the lower or upper limit, and the effect estimates were robust and reliable. There was also no publication bias according to Begg’s test (TC: *P* = 1.00; TG: *P* = 0.681, LDL-c: *P* = 0.392, HDL-c: *P* = 0.938) and Egger’s test (TC: *P* = 0.979; TG: *P* = 0.913, LDL-c: *P* = 0.509, HDL-c: *P* = 0.890), as shown in Supplementary Data Sheet 1.

### Hepatic enzymes ALT, AST, GGT, and ALP

**FIGURE 6:**
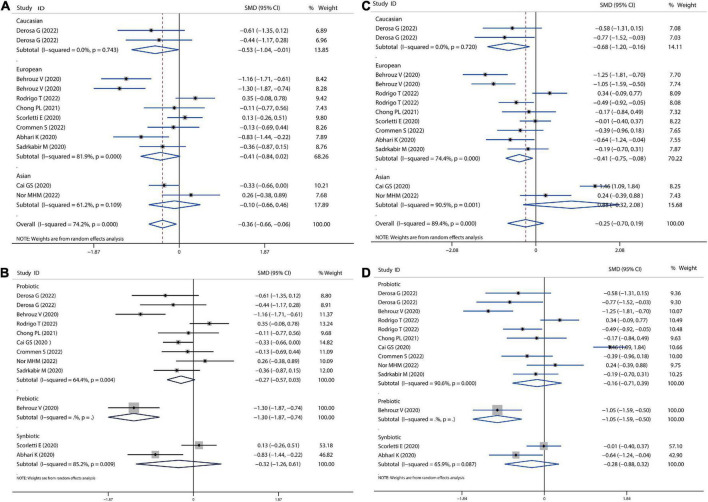
Forest plot of the effect of microbiota therapies on ALT and AST in patients with NAFLD. **(A)** The effect of microbiota therapies on ALT based on different ethnicity. **(B)** The effect of microbiota therapies on ALT based on different interventions. **(C)** The effect of microbiota therapies on AST based on different ethnicity. **(D)** The effect of microbiota therapies on AST based on different interventions. For each study, the estimated mean changes and the 95% CI are plotted with a diamond and a horizontal line, respectively.

**FIGURE 7:**
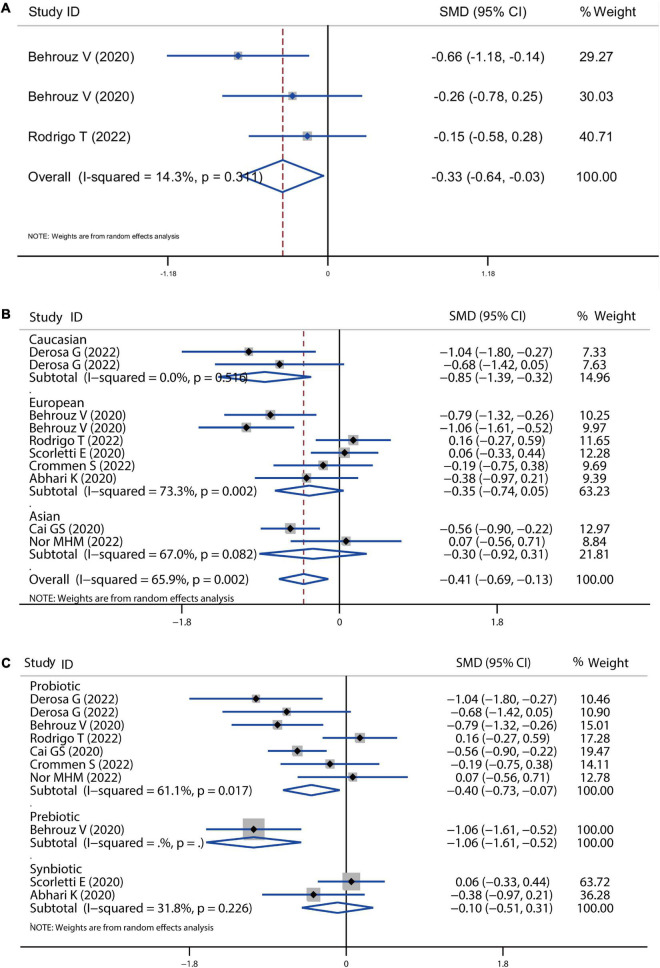
Forest plot of the effect of microbiota therapies on ALP and GGT in patients with NAFLD. **(A)** The total effect of microbiota therapies on ALP. **(B)** The effect of microbiota therapies on GGT based on different ethnicity. **(C)** The effect of microbiota therapies on GGT based on different interventions. For each study, the estimated mean changes and the 95% CI are plotted with a diamond and a horizontal line, respectively.

In our meta-analysis, all the treatments improved NAFLD disease by lowering levels of liver enzymes, including ALT, AST, ALP, and GGT, as shown in Figures 6, 7. All probiotics, prebiotics, or synbiotics treatments could reduce ALT (SMD = −0.36, 95% CI = −0.66 to −0.06, *I*^2^ = 74.2%, *P* = 0.046) (Figure 6A). The treatment effect presented more pronounced in Caucasian population (SMD = −0.53, 95% CI = −1.04 to −0.01, *I*^2^ = 0.0%, *P* = 0.063) than in European (SMD = −0.41, 95% CI = −0.84 to −0.02, *I*^2^ = 81.9%, *P* = 0.722) and Asian population (SMD = −0.10, 95% CI = −0.66 to −0.46, *I*^2^ = 61.2%, *P* = 0.018). Probiotics reduced ALT levels with a SMD of −0.27 (*P* = 0.083), and synbiotics reduced ALT with a SMD of −0.32 (*P* = 0.499) (Figure 6B), while prebiotics significantly reduced ALT (SMD = −1.30, 95% CI = −1.87 to −0.74, *P* < 0.001) reported by Behrouz et al. (18). Nevertheless, all treatments resulted in a non-significant reduction in AST levels (Figures 6C,D). In addition, all treatments were significantly effective for Caucasian (SMD = −0.68, 95% CI = −1.20 to −0.16, *I*^2^ = 0.0%, *P* < 0.001) and European population (SMD = −0.41, 95% CI = −0.75 to −0.08, *I*^2^ = 74.4%, *P* < 0.001), as displayed in Figure 6C.

In addition, all the treatments improved NAFLD disease by reducing ALP and GGT levels. As shown in Figure 7A, three comparisons showed the absolute change of ALP level after treatment with a significant SMD of −0.33 (*P* = 0.03). The level of GGT was also significantly decreased by all the treatments with an overall SMD of −0.41 (*P* = 0.004), which was lower induced by the treatment in the Caucasian population (SMD = −0.41, 95% CI = −0.75 to −0.08, *I*^2^ = 74.4%, *P* < 0.001) than European and Asian population (Figure 7B). The pooled effect of probiotic, prebiotic and symbiotic was −0.40 (*P* = 0.016), −1.06 (*P* < 0.001), −0.10 (*P* = 0.635), respectively (Figure 7C).

Sensitivity analysis showed no data outside of the lower or upper limit, and the effect estimates were robust and reliable. There was also no publication bias according to Begg’s test (ALT: *P* = 0.337, AST: *P* = 0.222, ALP: *P* = 0.117, GGT: *P* = 0.621) and Egger’s test (ALT: *P* = 0.305, ALP: *P* = 0.4556, GGT: *P* = 0.636), as shown in Supplementary Data Sheet 1.

### Fibrosis, steatosis, and systematic inflammation

**FIGURE 8:**
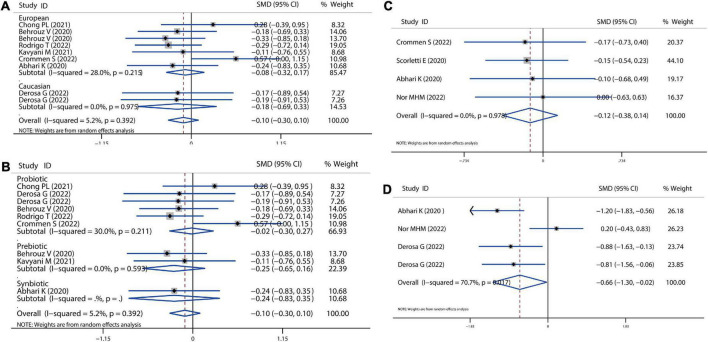
Forest plot of the effect of microbiota therapies on fibrosis, steatosis, and systematic inflammation in patients with NAFLD. **(A)** The effect of microbiota therapies on hs-CRP based on different ethnicity. **(B)** The effect of microbiota therapies on hs-CRP based on different interventions. **(C)** The effect of microbiota therapies on hepatic fibrosis. **(D)** The effect of microbiota therapies on hepatic steatosis. For each study, the estimated mean changes and the 95% CI are plotted with a diamond and a horizontal line, respectively.

There were four studies that utilized the hepatic fibrosis score and hepatic steatosis score to evaluate the NAFLD progression, with a non-significant reduction with a combined SMD value of −0.12 (*P* = 0.632), as depicted in Figures 8C,D. In addition, the probiotic and symbiotic treatment could also improve hepatic steatosis by combining analysis of four comparisons (SMD = −0.66, 95% CI = −1.30 to −0.02, *I*^2^ = 70.7%, *P* < 0.05). Furthermore, NAFLD can progress into non-alcoholic steatohepatitis (NASH), where inflammation and hepatocellular damage are associated with steatosis (11). In the present meta-analysis, there are nine comparisons related to the level of hs-CRP and showed a slight decrease without any significance after treatment (SMD = −0.10, 95% CI = −0.30 to 0.10, *I*^2^ = 5.2%, *P* = 0.313), as shown in Figures 8A,B.

## Discussion

The present meta-analysis indicated that microbial therapies ameliorate hepatic steatosis by reducing the levels of lipid profiles (TC, TG, LDL-c, HDL-c), liver enzymes (ALT, AST, ALP, and GGT), and HOMAI-R. Probiotics showed better efficacy than prebiotics and symbiotics. Moreover, the efficacy of probiotics in Caucasian population was proven to be better than that in the European or Asian population population. Nevertheless, probiotics, prebiotics, or synbiotics supplementation failed to improve BMI parameters, lifestyle (energy, carbohydrate, and fat intake), FBS, HbA1c, hs-CRP, and hepatic fibrosis in any population. Multiple factors, such as design, patient sex, genetics, dietary habits, lifestyle, or environmental strains may have contributed to each response to microbial therapies in the included studies.

Although multiple treatments have been used to improve or prevent NAFLD disease, a healthy lifestyle is always the most common and effective intervention for NAFLD. For example, a body mass loss of 3–5% could decrease cardiovascular risk, a body mass loss of 2–5% lower the level of HbA1c in T2DM patients, and a body mass loss of 7–10% could improve insulin sensitivity, liver enzyme concentrations, and liver steatosis and fibrosis in obese NAFLD patients (10, 31). Researchers investigated the BMI, and dietary habits (energy, carbohydrate, and fat intake) of NAFLD patients in the included trials. Previous studies found that probiotic supplementation could help weight loss and reduce body fat mass and waist circumference in overweight individuals to improve body composition and fat distribution (32). In addition, previous reports found that *B. coagulans* supplementation as a probiotic could produce short-chain fatty acids (SCFAs) (15). The binding of SCFAs to specific G-protein-coupled receptors (GPCRs) could stimulate the release of glucagon-like peptides (GLP-1), GLP-2, and PYY to maintain energy homeostasis and enhance fat storage (33, 34). However, when we analyzed the change in BMI after microbial therapies using data from nine RCTs, we found no improvement in the BMI of NAFLD patients. Meantime, the intake of energy, carbohydrate, and fat in the meal of these individuals remain unchanged. However, our results were still consistent with previous meta-analyses (20–22).

Previous findings indicated that probiotics intake promoted to decrease in fasting blood glucose in patients with type 2 Diabetes Mellites (T2DM) (35). Probably, probiotics contain multiple different microbial species that may affect the progress of sugar digestion and absorption, incretin secretion, and fat absorption (36, 37). In this study, we didn’t find any significant changes in FBS, insulin, and HbA1c in patients with NAFLD, which is in accordance with the previous result of the meta-analysis (21). In contrast, the change in HOMA-IR was statistically significant between microbial therapies and control subjects (*P* < 0.05). Chong et al. found that insulin resistance and liver inflammation are closely linked, especially HOMA-IR and AST (30). Moreover, a similar relationship between insulin sensitivity and AST and ALT has also been reported in the Insulin Resistance Atherosclerosis Study (IRAS) study (38). In our analysis, liver enzymes, including AST, ALT, ALP, and GGT, were significantly reduced by microbial therapies. In general, the imbalanced gut microbiota increases intestinal epithelial barrier permeability, and then various harmful substances, such as metabolites, lipopolysaccharide (LPS), and bacteria, bacterial DNAs will influx into the liver (39). Meantime, metabolites produced by an imbalanced microbiome, such as SCFAs, and bile acids, interact with mitochondrial function or genes, or influence the level of inflammatory factors and promote NAFLD process (40). Probably, supplementation of probiotics, prebiotics, or synbiotics corrected the imbalanced microbiota and improved the impaired liver, followed by decreased the levels of AST, ALT, ALP, and GGT.

The imbalanced gut microbiota also resulted in the activation of specific and non-specific immune responses between the intestinal tract and liver, following increased intestinal permeability, which may result in body blood lipid metabolism disorder (41). Microbial therapies improved intestinal flora disorder, and the metabolites of lactobacillus inhibit cholesterol synthase from regulating cholesterol. Simultaneously, the intestinal bacteria can also combine with cholesterol synthase, inhibit its absorption, and promote its excretion by influencing the circulation of cholesterol supplements in bile salts (42). This efficacy of probiotics has been demonstrated in the present meta-analysis. Microbial therapies could significantly decrease the lipid profiles, such as TC, TG, LDL-c, and HDL-c. In addition, the improvement of lipids is closely associated with the stimulation of adenosine 50-monophosphate (AMP)-activated protein kinase (AMPK) and serine/threonine kinase (AKT) proteins, and lipogenesis- or lipolysis-related proteins induced by microbial supplementation (43). Previous research also revealed that probiotics could decrease the number of duodenal CD4 + and CD8 + T lymphocytes to affect the mucosal immune function to improve lipid metabolism dysregulation (44).

Microbial therapies also could be a potential target for local mucosal inflammation, such as hs-CRP, IL-8, and TNF-alpha (11, 45). Previous research indicated that supplementation with *Bifidobacterium* long with fructooligosaccharides significantly reduced serum hs-CRP levels in NASH patients (18). Probably, intestinal flora disorder changed by the microbial intervention, subsequently, SCFA production was increased, which reduced the expression of inflammation-relevant genes (46). On the other hand, SCFAs products can also increase the level of IL-18 expression, which is associated with the decreased enzymatic synthesis of hepatic CRP (47). There were also RCTs showing no significant change in hs-CRP after the microbial intervention. For example, Chong PL, et al performed VSL^#^3^®^ probiotic supplementation for NAFLD patients (30), and Crommen et al. performed a specifically tailored multistrain probiotic supplementation for NAFLD patients (10). We combined analysis of the eight included studies and found that microbial intervention failed to improve the level of hs-CRP. Nevertheless, we found an improvement in liver steatosis even without a change in systematic inflammation. In general, inflammation and hepatocellular damage are associated with steatosis during NAFLD progressing into NASH (48).

## Limitations

This meta-analysis had some limitations despite the fact that we had demonstrated significant improvement in liver enzymes, lipid profiles, HOMA-IR, and liver steatosis after microbial therapies. First, the included RCTs had a small sample size, diverse populations and regions, and different drug treatments and diets, which will generate heterogeneity and influence the stability of the combined results. Therefore, we conducted the subgroup meta-analysis according to population and intervention. However, other subgroups couldn’t be performed become of the absence of detailed data. Second, sex hormones and chromosomes have a definite impact on the differences in microbiomes between men and women (49). A previous study revealed that a sex-specific microbiome might play a critical role in NAFLD and obesity incidence (50). Nevertheless, the included RCTs didn’t involve any subgroup according to sex, and microbial therapies may generate different improvements in men or women with NAFLD, which may lead to the heterogeneity of the intervention results. Third, we transformed the median, or the first and third quarter values, into the mean and SD values at both baseline and final points of the formula, which may also generate error and bias. Fourth, the duration of the included studies ranged from 2.5 to 14 months, which may influence the effectiveness of treatment and result in the instability of the combined analysis.

## Conclusion

The present meta-analysis focuses on the clinical efficacy of microbial therapies for NAFLD. Supplementation probiotics, prebiotics, or symbiotic may improve glucose homeostasis, decrease blood lipid, and improve liver enzymes and hepatic steatosis in patients with NAFLD. Moreover, probiotics was more effective in improving NAFLD in the Caucasian population than prebiotics or symbiotic in the Asian or European populations. Nevertheless, our results didn’t show any significant effect of microbial therapies on BMI, FBS, hs-CRP, and hepatic fibrosis in patients with NAFLD. In the future, more studies that considering patients’ sex, strains, sample size, and duration should be performed on NAFLD patients under RCT design and multiple centers.

## Author contributions

WX and ZYZ were responsible for the design. WG, GM, and XD were responsible for writing of this work. ZLZ and XL performed the literature search. WX and XL were responsible for the data extraction. XD was responsible for the data analysis. All authors reviewed this draft and approved the final manuscript.

## Footnotes

1
https://community.cochrane.org/help/tools-and-software/revman-5

## Supplementary material

Supplementary Figure 1 Risk of bias in the included RCTs evaluating probiotic, prebiotic, and symbiotic for treatment of NAFLD.
