# Supplementary material for: The effects of supplementation of probiotics, prebiotics, or synbiotics on patients with non-alcoholic fatty liver disease: A meta-analysis of randomized controlled trials
Source: Front Nutr. 2022 Oct 25;9:1024678. doi: 10.3389/fnut.2022.1024678 (PMC9640999; doi:10.3389/fnut.2022.1024678)

1. ALP influence analysis

ALP publication bias

1. ALT influence analysis

ALT publication bias

1. AST influence analysis

AST publication bias


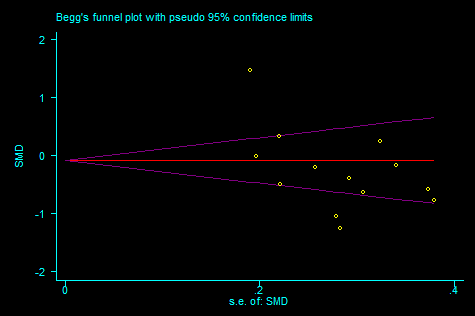


1. BMI influence analysis


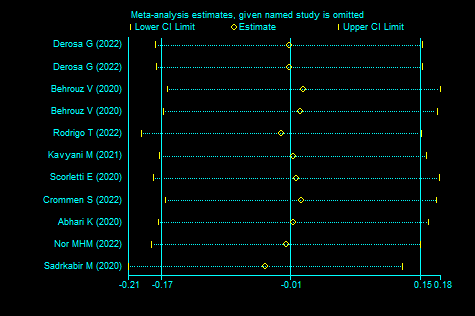


BMI publication bias


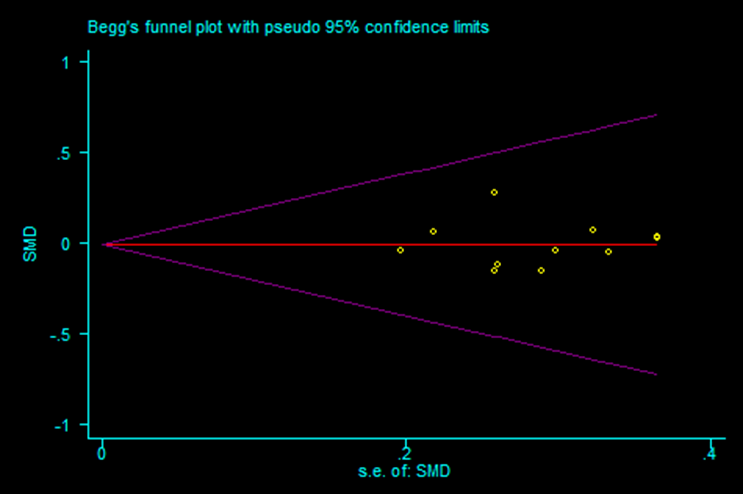


1. fat influence analysis


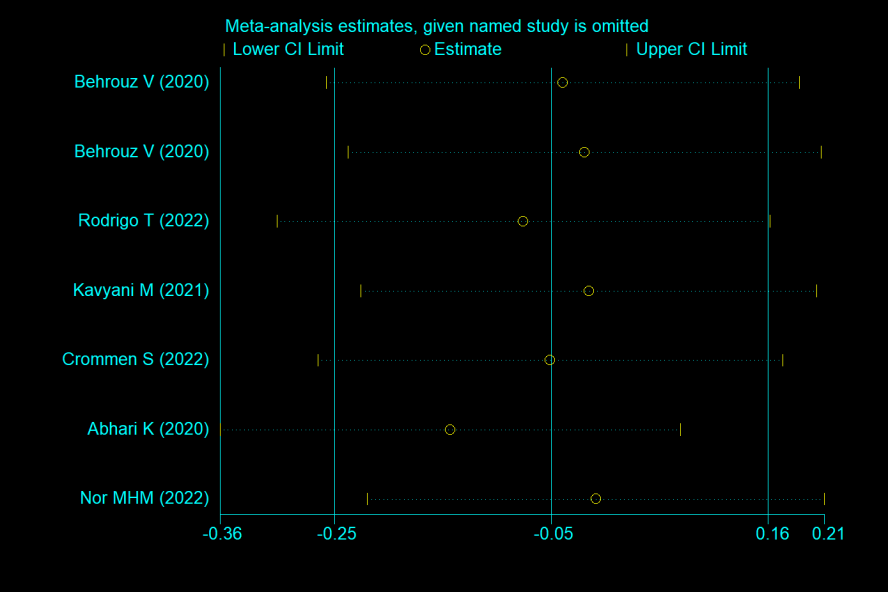


fat publication bias


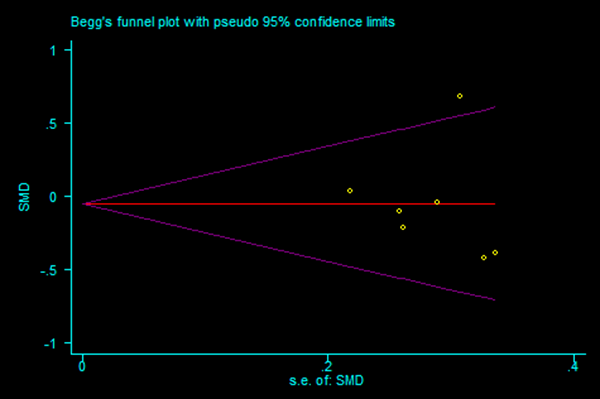


1. energy influence analysis


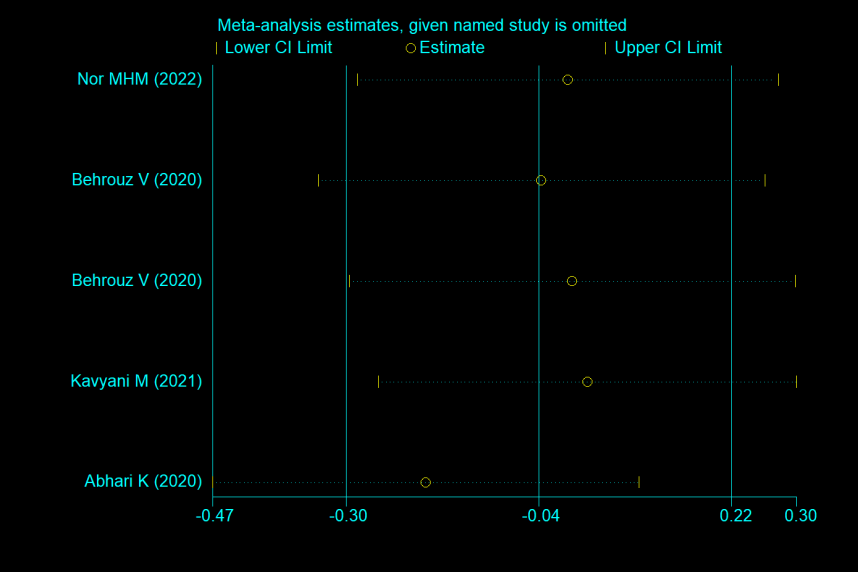


Energy publication bias


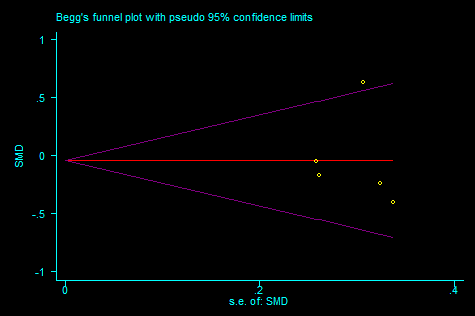


1. FBS influence analysis


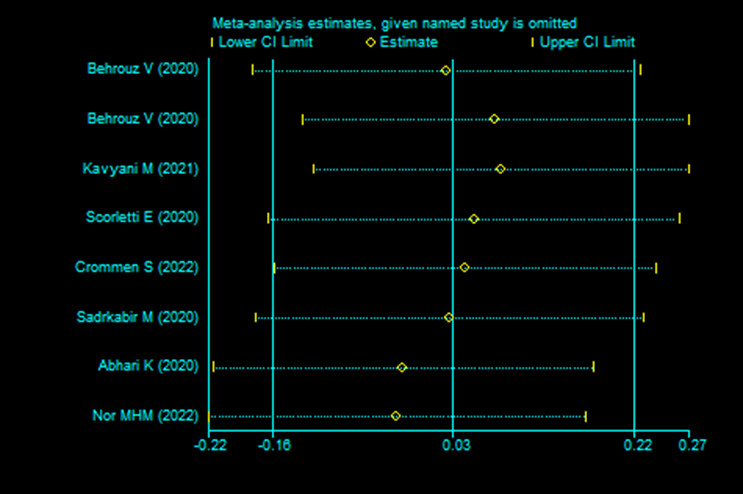


FBS publication bias


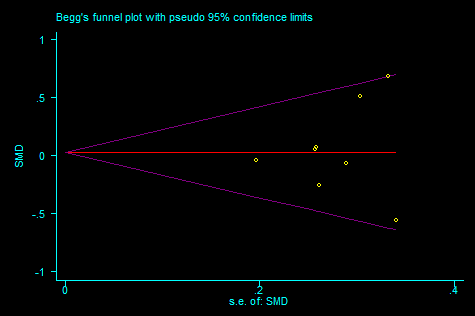


1. GGT influence analysis


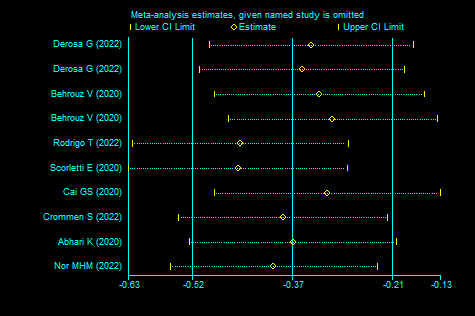


GGT publication bias


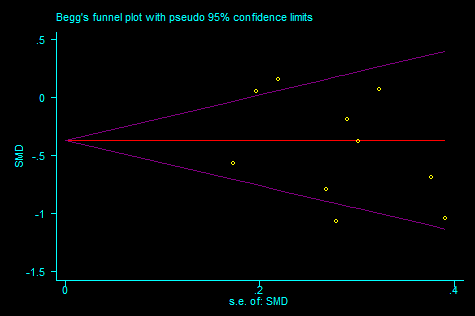


1. HDL influence analysis


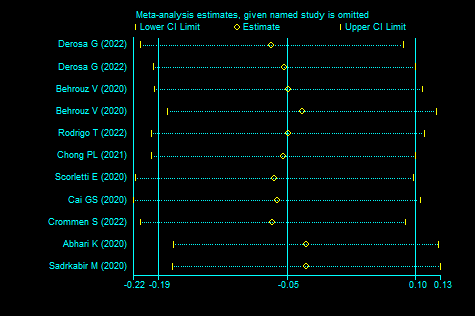


HDL publication bias


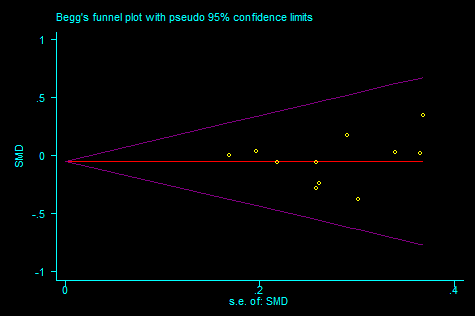


1. HOMIA-IR influence analysis


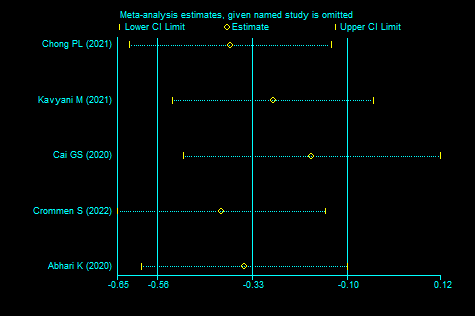


HOMIA-IR publication bias


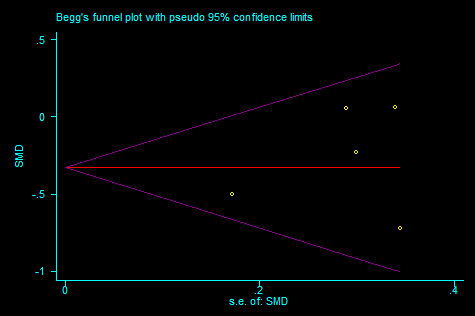


1. Hs-CRP influence analysis


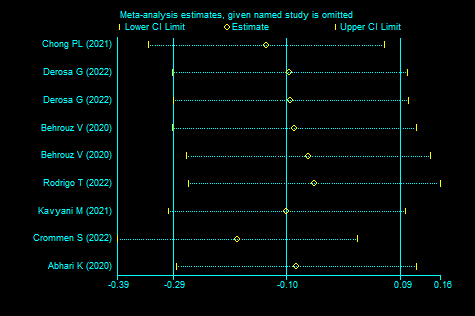


Hs-CRP publication bias


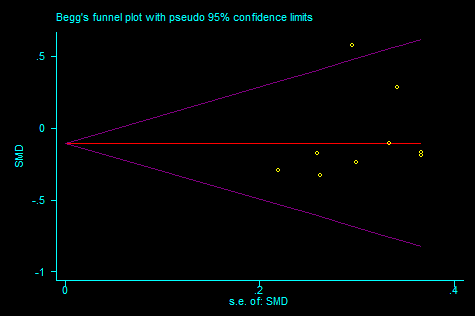


1. Insulin influence analysis

**
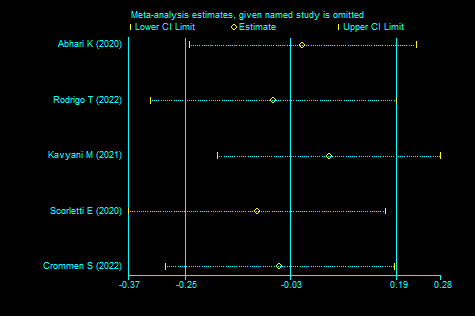
**

Insulin publication bias

**
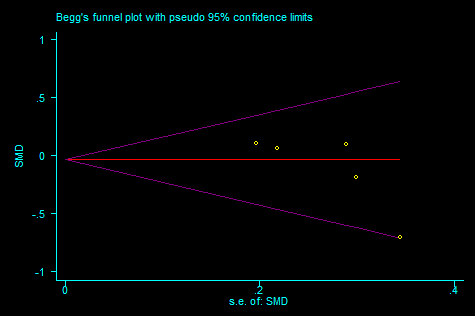
**

1. LDL influence analysis


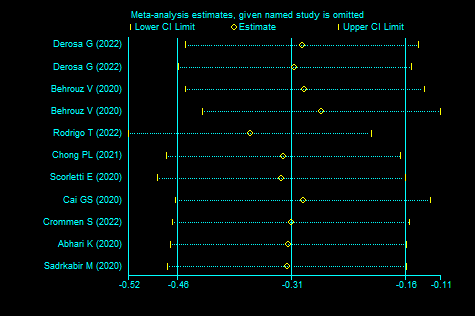


LDL publication bias


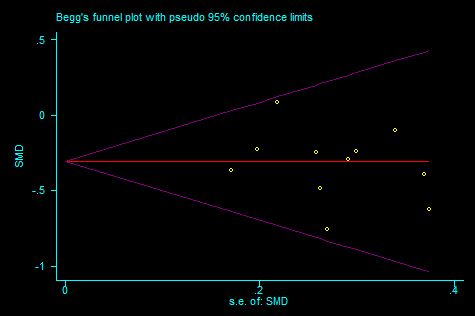


1. TC influence analysis


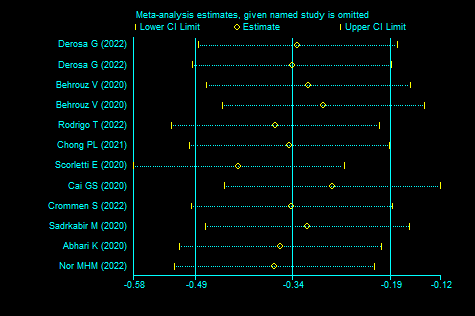


TC publication bias


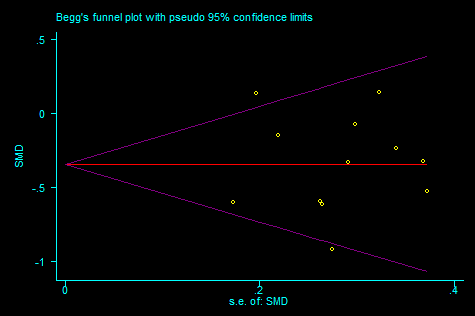


1. TG influence analysis


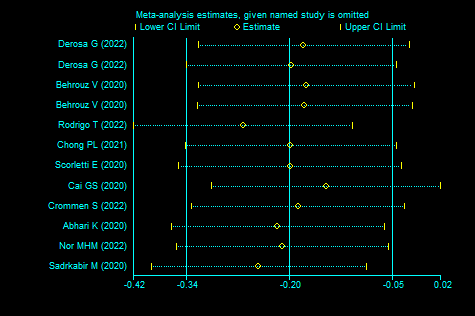


TG publication bias


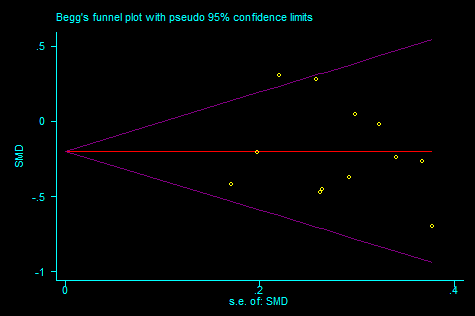

Supplement: Supplementary file 1 [file Data_Sheet_1.docx]
