# Supplementary figures and images for: The effects of supplementation of probiotics, prebiotics, or synbiotics on patients with non-alcoholic fatty liver disease: A meta-analysis of randomized controlled trials
Source: Front Nutr. 2022 Oct 25;9:1024678. doi: 10.3389/fnut.2022.1024678 (PMC9640999; doi:10.3389/fnut.2022.1024678)

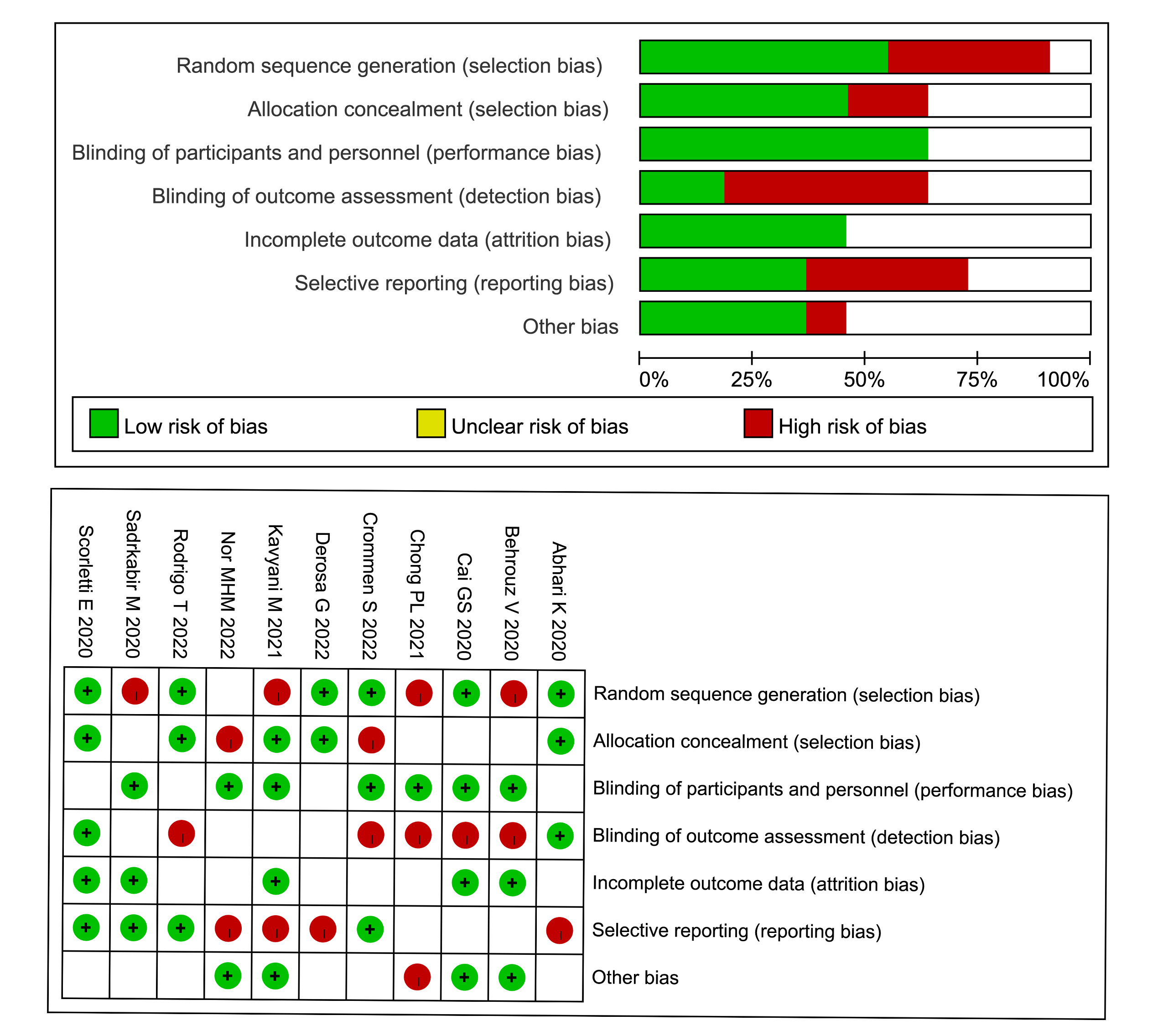

Supplement: Supplementary Figure 1 — Risk of bias in the included RCTs evaluating probiotic, prebiotic, and symbiotic for treatment of NAFLD. [file Image_1.TIF]
